# The ADMR Receptor Mediates the Effects of Adrenomedullin on Pancreatic Cancer Cells and on Cells of the Tumor Microenvironment

**DOI:** 10.1371/journal.pone.0007502

**Published:** 2009-10-22

**Authors:** Vijaya Ramachandran, Thiruvengadam Arumugam, Robert Langley, Rosa F. Hwang, Pablo Vivas-Mejia, Anil K. Sood, Gabriel Lopez-Berestein, Craig D. Logsdon

**Affiliations:** 1 Department of Cancer Biology, University of Texas M. D. Anderson Cancer Centre, Houston, Texas, United States of America; 2 Department of Medical Oncology, University of Texas M. D. Anderson Cancer Centre, Houston, Texas, United States of America; 3 Department of Surgical Oncology, University of Texas M. D. Anderson Cancer Centre, Houston, Texas, United States of America; 4 Department of Gynecologic Oncology, University of Texas M. D. Anderson Cancer Centre, Houston, Texas, United States of America; 5 Department of Experimental Therapeutics, University of Texas M. D. Anderson Cancer Centre, Houston, Texas, United States of America; 6 Center for RNAi and Non-Coding RNA, University of Texas M. D. Anderson Cancer Centre, Houston, Texas, United States of America; Deutsches Krebsforschungszentrum, Germany

## Abstract

**Background:**

Adrenomedullin (AM) is highly expressed in pancreatic cancer and stimulates pancreatic cancer cells leading to increased tumor growth and metastasis. The current study examines the role of specific AM receptors on tumor and cells resembling the tumor microenvironment (human pancreatic stellate - HPSC, human umbilical vein – HUVEC and mouse lung endothelial cells - MLEC).

**Methods and Findings:**

AM receptors ADMR and CRLR were present in HPSC, HUVEC and MLECs while PDAC cells possessed only ADMR receptors as assessed by RT-PCR and western blotting. All cell lines expressed and secreted AM as indicated by ELISA. The growth of each of the cell lines was stimulated by exogenous AM and inhibited by the antagonist AMA. AM also stimulated *in vitro* angiogenesis assessed by polygon formation of endothelial cell lines. SiRNA-mediated silencing of ADMR, but not CRLR, reduced basal growth of all cells examined and reduced polygon formation of endothelial cells *in vitro*. Orthotopic tumors developed with shADMR bearing cancer cells had dramatically reduced primary tumor volume (>90%) and lung and liver metastasis compared to shControl bearing cells. To validate ADMR as a potential therapeutic target, i*n vivo* studies were conducted using neutral nanoliposomes to systemically deliver human siRNA to ADMR to silence human cancer cells and mouse siRNA to ADMR to silence mouse tumor stromal cells. Systemic silencing of both human and mouse ADMR had no obvious adverse effects but strongly reduced tumor development.

**Conclusion:**

ADMR mediates the stimulatory effects of AM on cancer cells and on endothelial and stellate cells within the tumor microenvironment. These data support the further development of ADMR as a useful target treatment of pancreatic cancer.

## Introduction

Pancreatic cancer is the fourth leading cause of cancer-related death in the United States and it has been estimated that in 2008 approximately 37,680 Americans were diagnosed and 34,290 died from this disease [1]. Although significant advances are beginning to be made into the management of the disease, the 5-year survival rate has not improved over the past 25 years [1]. The high mortality rate is due to the high incidence of metastatic disease at initial diagnosis, the aggressive clinical course and the failure of systemic therapies [2]. Therefore, there is an urgent need for improved understanding of the molecular biology of the disease that can be utilized to develop new therapies for pancreatic cancer.

We previously showed that adrenomedullin (AM) is over-expressed in pancreatic cancer and has a strong autocrine role in this disease [3]. AM is a 52 amino acid peptide originally isolated from human pheochromocytoma [4] that acts as a multifunctional regulatory peptide [5]. One issue with AM as a target for cancer therapy is that AM has several physiological functions whose inhibition may be detrimental. AM is expressed in normal pancreatic islet cells, with predominant expression in the F cells, which also contain pancreatic polypeptide [6]. AM reduces insulin secretion in physiological conditions [6]. AM also has important effects in vascular cell biology where it regulates vascular tone and permeability and promotes vasodilation [7]–[11]. AM is also a potent angiogenic molecule, especially in hypoxia, which induces AM [10]. The angiogenic effects of AM are likely mediated through direct stimulation of endothelial cell proliferation [12] and protection of endothelial cells from apoptosis [13]. Adrenomedullin signaling is necessary for murine lymphatic vascular development [14]. Mice in which AM has been genetically deleted develop cutaneous edema and midgestational lethality due to defect in lymphatic vessel growth and cardiovascular defect [15]. In cancer, AM appears to have an important role in angiogenesis as well as an additional trophic effect directly on cancer cells [16]–[18].

AM acts as a peptide ligand that activates receptors on the cell surface. Pancreatic beta cells express receptors known to respond to AM including the adrenomedullin receptor (ADMR, also known as L1-R) and the calcitonin-receptor-like-receptor (CRLR) [6], [19], [20]. Our previous study found that pancreatic cancer cells express only ADMR, while both receptors are present in cells found within the tumor microenvironment including human pancreatic stellate cells (HPSCs) and endothelial cells. However, the roles of the specific receptors in AM's effects on pancreatic cancer, HPSCs and endothelial cells are currently unknown.

The current study examines the effects of AM on human pancreatic tumor cells, HPSCs and endothelial cells and investigates the receptors involved in these effects. We silenced each of the receptors *in vitro* using siRNA and found that ADMR was primarily responsible for the biological effects of AM on each of these cell types. We then examined the effects of silencing ADMR on pancreatic cancer cells and observed a major reduction of tumor growth *in vivo*. To analyze the potential of ADMR as a target of cancer therapy, we then evaluated the effects of silencing ADMR in both mouse cells that make up the tumor microenvironment and on human cancer cells by using DOPC nanoliposomes to deliver species specific siRNAs. This systemic silencing of ADMR did not have obvious deleterious effects on healthy mice but greatly reduced tumor development. Therefore, ADMR should be an important target for the future development of small molecule therapeutics.

## Materials and Methods

### Cell Lines and AM Peptides

Pancreatic cancer BxPC3 cells and HUVEC cell lines were obtained from the American Type Culture Collection (Manassas, VA). MPanc96 pancreatic adenocarcinoma cell lines were originally established by Dr. Timothy J. Eberlein (St. Louis, MO) [21]. MLECs are microvascular endothelial cells from primary cultures obtained from lungs of mice whose tissues harbor a temperature-sensitive SV40 large T antigen [22]. When cultured under permissive temperatures (33°C), cell lines displayed doubling times consistent with endothelial cells possessing an angiogenic phenotype. The transfer of these endothelial cells to non-permissive temperatures (37°C) resulted in cell differentiation and the induction of a quiescent state. MLECs were cultured in 10% FBS in DMEM. BxPC3 cells were cultured in 10% FBS in RPMI media, MPanc96 cells were cultured in 10% FBS in DMEM media and human endothelial cells (HUVEC) were cultured in 15% FBS in MEM. All media contained 1% antibiotic. Human pancreatic stellate cells (HPSCs) were isolated using the outgrowth method from pancreatic adenocarcinoma samples from patients undergoing surgical resection and cultured in 15% FBS in DMEM [3], [23]. All cells were maintained at 37°C in a humidified atmosphere of 5% CO_2._ BxPC3 and MPanc96 cell lines stably bearing shControl or shADMR vectors and the luciferase gene developed in an earlier study were also used [3]. Adrenomedullin (AM 52) and adrenomedullin antagonist (AMA (AM 22–52)) were purchased from Sigma, (St. Louis, MO).

### ELISA for AM

AM was detected in conditioned media from HPSC, HUVEC and MLECs using a commercial ELISA. For collection of conditioned media, cell lines were grown to 80% confluence and washed with PBS and then cultured for 24 hrs in their respective serum-free media. Media was collected and concentrated using Centricon YM-3 filter devices (Millipore Corporation, Chicago, IL). Protein concentrations were determined using Bio-Rad reagent (Bio-Rad Laboratories, Hercules, CA). Competitive ELISA was then conducted using an AM detection kit (Phoenix Pharmaceuticals, Belmont, CA; cat # EK-010-01) following the manufacturer's suggested protocol. Respective concentrated serum-free media served as reagent control and control OD values were subtracted from those of all other samples. Calculations of AM concentration utilized the standard curve included in the kit and were conducted according to the manufacturer's protocol.

### Transient transfection of siRNA

Silencing of ADMR and CRLR was achieved in HPSC, HUVEC and MLECs (2×10^4^ cells) using transient transfection of siRNAs. Human and mouse siRNAs each against control, ADMR and CRLR (siRNAs ID # 4611, 46184, 42272, Ambion Inc. Austin, TX and custom made mouse siRNAs ID # Control - 1129089, 1129090; ADMR - 1129085, 1129086; CRLR - 1129087, 1129088 from Sigma-Aldrich (Proligo), St.Louis, MO) were transfected at a final concentration of 5 nM per well on 96-well plates. We also analyzed the effects of siADMR on MPanc96 cancer cells. SiRNA transfection was carried out with Hiperfect transfection reagent (Qiagen, Valencia, CA) as per the manufacturer's protocol.

### Western Blotting

MPanc96, HPSC, HUVEC and MLEC cells were transiently transfected with control siRNA or siRNAs against ADMR or CRLR and after 72 hours, cell lysates were prepared and protein concentrations were measured by BioRad reagent. Protein (50 µg) was loaded onto 10% SDS-PAGE gels and western blotting was conducted using a primary antibody against ADMR (cat # LS-A4048 MBL International Corporation, Woburn, MA) at a 1: 1000 dilution and using a primary antibody against CRLR (cat # sc-30028, Santa Cruz Biotechnology, Santa Cruz, CA) at a 1: 100 dilution. The same blot was re-probed for β-Actin (1∶200 dilution; cat # A2066 Sigma, St.Louis, MO), which served as loading control.

### RT-PCR

Total RNA was extracted from HPSC, HUVEC and MLEC cells with or without siRNA transfection and from mouse pancreas. DNase was used to remove contaminating genomic DNA after RNA purification. The integrity of the RNA was confirmed by running on a denaturing gel, and observing clear 28S and 18S rRNA bands. A non-reverse transcribed control was run to assure that no genomic DNA was amplified. RT-PCR was conducted with human and mouse AM/ADMR/CRLR primers to determine their expression and levels of silencing. Reverse transcription was followed by 35 cycles of standard PCR (1-min denaturation at 94°C, 1-min annealing at 63°C, and 1-min extension at 72°C). Primers designed for human AM (Genebank: NM_001124) were: forward 5′CGG GAT CCA TGA AGC TGG TTT CCG TC 3′ and reverse, 5′ CGG AAT TCC TAA AGA AAG TGG GGA GC 3′. Primers designed for human ADMR (Genebank: NM_007264) were: forward 5′ CAT CGC GGA CCT GGG CAT TGT 3′ and reverse, 5′ TGA GAG GGA AGG GCA GCA GGA AGC 3′. Primers designed for human CRLR (Genebank: NM_005795) were: forward 5′TGC TCT GTG AAG GCA TTT AC 3′ and reverse, 5′ CAG AAT TGC TTG AAC CTC TC 3′. Primers designed for mouse AM (Genebank: NM_009627) were:forward 5′ CCA GGG TTC CCG CAG CAA 3′ and reverse, 5′ CTA TAT CCT AAA GAG TCT GG 3′. Primers designed for mouse ADMR (Genebank: NM_007412) were: forward 5′ CCG TTA CCT TCC CAA GGA 3′ and reverse, 5′ TTA GCT GGC TAC AGA ATT GCA 3′. Primers designed for mouse CRLR (Genebank: NM_018782) were: forward 5′ TGG CTT TTC CCA CTC TGA T 3′ and reverse, 5′ TCA CAT CAC TAG ATC ATA CGT 3′. 18S primers served as loading control for the RT-PCR reactions. Amplified products were separated on 1.5% agarose gels and visualized by ethidium bromide.

### Cell growth studies

HPSC, HUVEC and MLECs (1000 cells) were plated on 96-well plates in 0.5% serum containing media with or without AM (0–200 nM) or AMA (1 µM), which were refreshed daily, and cell numbers were estimated after 48 hours for HPSC and HUVEC cells and after 96 hours for MLECs by MTS assay. For siRNA studies, HPSC, HUVEC and MLECs (1000 cells) were plated on 96-well plates and respective human and mouse siControl/siADMR/siCRLR were transfected and cell numbers were estimated after 48 hours for HPSC and HUVEC cells and after 96 hours for MLECs. Cell growth was analyzed using the MTS reagent added one hour before taking the spectrophotometric reading according to the manufacturer's directions (Promega, Madison, WI).

### *In vitro* Angiogenesis assay

HUVEC and MLEC (1×10^4^) cells were seeded onto the surface of the polymerized ECMatrix prepared as described by the manufacturer (Cat # ECM625; Chemicon Intl, Millipore Corp, Billerica, MA). Cells were treated with AM (200 nM) or AMA (1 uM) peptides and after 6 hours tube formation was evaluated under an inverted light microscope (Olympus, Center Valley, PA). Images were captured using a chilled, charge-coupled device camera (Hamamatsu, Bridgewater, NJ) and SmartCapture software (Digital Scientific, Cambridge, UK). Images were further processed with Adobe Photoshop software (Adobe Systems, Mountain View, CA). To quantify the angiogenic events, the patterns of cell growth in 10 random fields at 100× magnification was assessed as per the manufacturer's suggestions and graded as: Individual cells, well separated –0; Cells migrated and aligned –1; Capillary tubes visible, no sprouting –2; Sprouting of capillary tubes –3; Closed polygons formed – 4; and complex mesh-like structures developed –5.

### Immunohistochemical staining - CD31/VEGF

Unstained 4 µm tissue sections from siControl or siADMR treated mice were deparaffinized with xylene and rehydrated with ethanol. Endogenous peroxidase activity was blocked with 3% hydrogen peroxide in methanol and non-specific binding sites were blocked with protein blocking solution (5% normal horse and 1% normal goat serum). Primary antibody against CD31 (1: 800 dilution; cat # 01951A; BD Pharmingen, San Diego CA)/VEGF (1∶100 dilution; cat # sc-152; Santa Cruz Biotechnology, Santa Cruz, CA) was added and samples were incubated overnight at 4°C. Secondary antibody was added and incubated for 1 hour at room temperature. Finally, slides were developed with 3,3-diaminobenzidine (DAB) substrate counterstained with hematoxylin, dehydrated with ethanol, fixed with xylene and mounted. Immunohistochemistry was analyzed using an inverted light microscope (Olympus, Center Valley, PA). Images were captured using a chilled, charge-coupled device camera (Hamamatsu, Bridgewater, NJ) and SmartCapture software (Digital Scientific, Cambridge, UK). Images were further processed with Adobe Photoshop software (Adobe Systems, Mountain View, CA). Slides were blinded and analyzed by IHC core in Dept. of Cancer Biology, UT MDACC.

#### DOPC nanoliposome coupled siRNAs

For experiments to test the efficacy of *in vivo* therapeutic targeting of ADMR in orthotopic tumors in mice, neutral liposomes containing siRNAs were prepared as previously described [24]. Briefly, siRNA oligonucleotides (without hairpins) to ADMR target sequences (human siADMR (sense - 5′ rGrCUrGrCUUrGrArCrCUrCUUrCrArArCTT 3′); mouse siRNA sense sequence – 5′ rCrCUUUUrGrArArArCrGUrArCrArGrCrGTT 3′) or control sequences (control siRNA sense sequence – 5′ UUrCUrCrCrGrArArCrGUrGUrCrArCrGUTT 3′) (custom made human and mouse oligos cat # Human siADMR - 1150080, 1150081; Mouse siADMR - 1129085-H, 1129086-H; Control - 1129089, 1129090 from Sigma-Aldrich (Proligo), St.Louis, MO) were mixed with 1,2-dioleoyl-sn-glycero-3-phosphatidylcholine (DOPC) (Avanti Polar Lipids, Alabaster, AL) at a ratio of 10∶1 (w/w) DOPC/siRNA and lyophilized. Immediately before *in vivo* administration, lyophilized preparations were hydrated in 0.9% saline at a concentration of 10 µg of siRNA per 200 µl and were purified by separating free siRNA from liposomes with filter units with a size exclusion limit of 30,000 Daltons (Millipore Corp, Billerica, MA).

#### Glucose Tolerance Test

For glucose tolerance, mice were injected intraperitoneally with 1.5 mg of glucose/g body weight at 9:00 a.m., after a 16-h fast. Blood glucose was determined at the indicated times with samples of tail blood obtained using the Ascensia CONTOUR Blood glucometer (Bayer Health Care, Tarry town, NY).

#### *In vivo* studies

All animals were handled in strict accordance with good animal practice as defined by the relevant national and/or local animal welfare bodies, and all animal work was approved by the appropriate committee (IACUC - 09-04-08832). ***Orthotopic tumor model.*** MPanc96 and BxPC3 cells bearing shADMR or shControl were developed as mentioned in a previous publication [3]. These pancreatic cancer cells were further modified to stably express the firefly luciferase gene by lentivirus transfection to facilitate *in vivo* monitoring of tumor development [25]. These cells were grown to 80% confluence, harvested by trypsinization, washed twice in PBS, and resuspended to a final concentration of 1×10^6^ cells/50 ul of MPanc96 cells and 2×10^6^ cells/50 ul of BxPC3 cells in sterile PBS and injected into the pancreas of four-week old male *athymic nude* mice (n = 5). Tumor growth was assessed by bioluminescence imaging at the end of four weeks. ***Lung and liver metastasis models.*** Comparisons were made between lung and liver metastasis in MPanc96 cells bearing shADMR or shControl cells using well established metastasis models. Lung metastases were developed by tail vein injection of 1×10^6^ cells/100 ul (n = 9) and liver metastases by splenic injection of 1×10^6^ cells/50 ul (n = 7) to *athymic nude* mice and evaluated by bioluminescence imaging. After six weeks, mice were sacrificed and organs were excised and stored in Bouin's fixative solution. ***Delivery of DOPC nanoliposome coupled siRNAs.*** For evaluation of potential toxicity, experiments were conducted delivering DOPC nanoliposome coupled siControl or siADMR (mouse) to *athymic nude* mice (10 ug per animal i.p. twice a week for four weeks). Water and food intake, body weight and blood glucose levels were measured. Animals were sacrificed after four weeks, the pancreas was isolated and total RNA was extracted. RT-PCR was used to evaluate the silencing of ADMR, while 18S served as internal control. **Orthotopic tumors** were developed in *athymic nude* mice with MPanc96 cells bearing the luciferase gene (1×10^6^ cells/50 ul of sterile PBS) and two weeks later, DOPC nanoliposome coupled siControl and siADMRs (human and mouse in combination) were delivered 10 ug per animal i.p. twice a week for six weeks. **Bioluminescence imaging**. Bioluminescence imaging was conducted using a cryogenically cooled imaging system coupled to a data acquisition computer running LivingImage software (Xenogen Corp., Alameda, CA). Before imaging, animals were anesthetized in an acrylic chamber with 1.5% isofluorane/air mixture and injected i.p. with 40 mg/ml of luciferin potassium salt in PBS at a dose of 150 mg/kg body weight. A digital grayscale animal image was acquired followed by acquisition and overlay of a pseudocolor image representing the spatial distribution of detected photons emerging from active luciferase within the animal. Signal intensity was quantified as the sum of all detected photons within the region of interest per second. After the final tumor imaging, the tissues were removed and the animals were re-imaged to visualize and count cancer cell dissemination and metastases. Tissues were also fixed with formaldehyde and histology was evaluated to verify the accuracy of the bioluminescence data.

### Statistical Analysis

All *in vitro* experiments were conducted in triplicate and carried out on three or more separate occasions. Data presented are means of the three or more independent experiments±Standard Error of the Mean (SEM). Statistically significant differences between controls and treated samples were determined by two-tailed unpaired Student's T-test and were defined as *p<0.05. Results were compared using GraphPad Prism 4 software (GraphPad Software, http://www.graphpad.com).

## Results

### AM has autocrine effects on HPSC, HUVEC and MLEC cells

**Figure 1:**
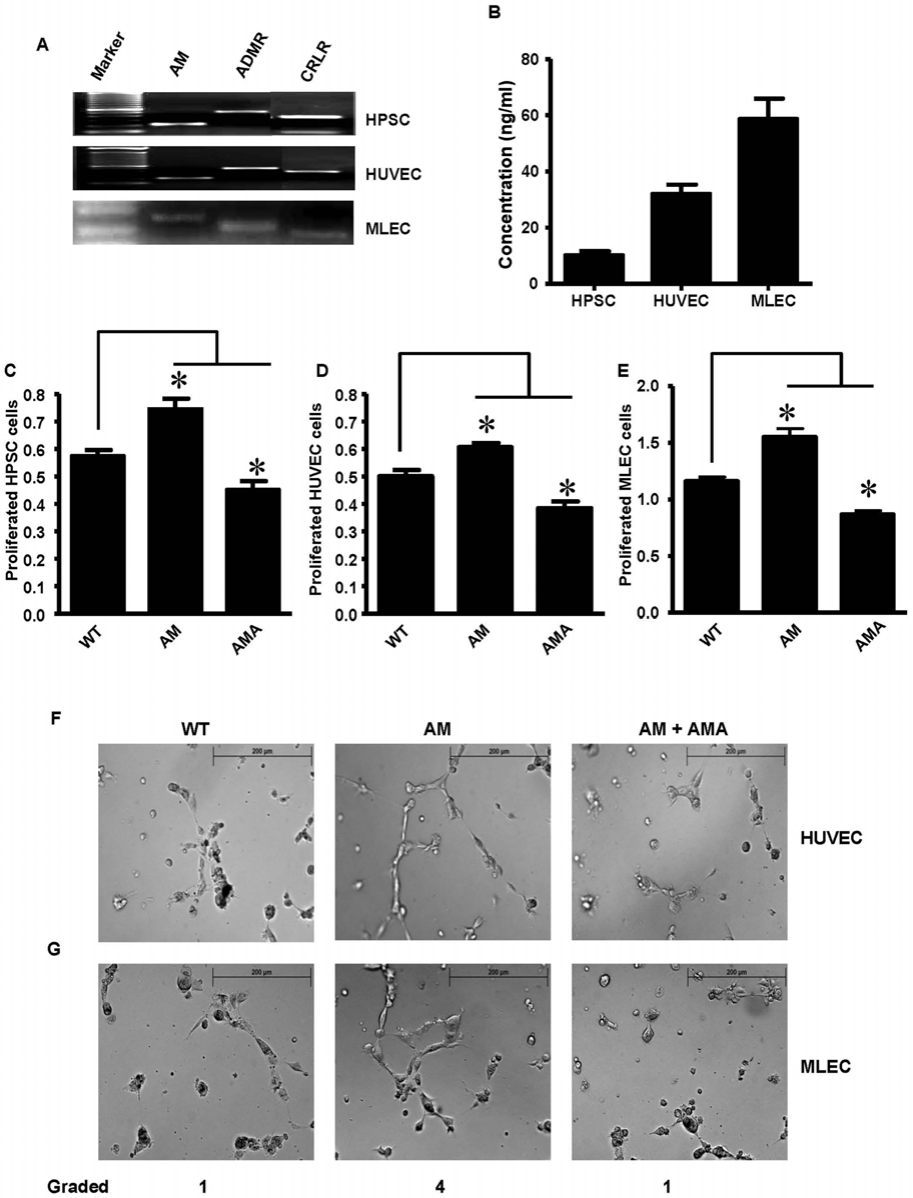
Autocrine effects of AM on HPSC and endothelial cells. (A) RT-PCR showing the expression of AM, ADMR, and CRLR on HPSC, HUVEC and MLECs. (B) ELISA assay showing the secretion of AM from HPSC, HUVEC and MLEC cells. Serum-free media bathing these cells were collected and concentrated and assayed for the presence of AM. Levels were calculated as per manufacturer's instruction. (C) HPSC, (D) HUVEC, or (E) MLEC cells (1000 cells) were plated on 96-well plates and AM (50 nM for HPSC and 200 nM for HUVEC and MLEC) or AMA (1 uM) were added to the plates daily and cell numbers were estimated after 48 hours for HPSC and HUVEC cells and after 96 hours for MLECs by MTS assay. When compared to the WT control group, AM stimulated the proliferation of all three cell types. Likewise, AMA treatment inhibited basal proliferation of all of the cells. Data shown are means +/− SEM (*p<0.05). *In vitro* angiogenesis assays were conducted with (F) HUVEC and (G) MLEC cells. Cells were plated on EC matrix along with AM (200 nM) alone or in combination with AMA (1 uM). After 6 hours tube formation was evaluated microscopically as per manufacturer's instruction. Shown are representative micrographs. AM consistently stimulated HUVEC and MLEC cells to form tubes, while AMA reversed the effects of AM.

Previously we observed that AM served as an autocrine regulator of proliferation, survival, and motility of pancreatic cancer cells [3]. To determine whether AM also influenced HPSC, HUVEC and MLECs, we first examined whether they expressed AM and its receptors ADMR and CRLR as measured by RT-PCR. All three cell lines expressed AM, ADMR and CRLR (Fig. 1A). Further, we assessed whether these cells secreted AM into tissue culture media using an ELISA. All these cell types secreted AM (Fig. 1B), supporting the role of this molecule as an autocrine regulator in multiple cell types. To examine the effects of AM on the biology of these cells, exogenous AM was added to HPSC (Fig. 1C), HUVEC (Fig. 1D) and MLEC (Fig. 1E) cells and proliferation was assessed by the MTS assay. Previous studies indicated that the optimal concentration of AM was 50 nM for HPSC and 200 nM for HUVEC and MLEC cells (data not shown). Addition of AM at the optimal concentrations stimulated the growth of HPSCs by 29±1.8% (p<0.05), HUVECs by 25±3.7% (p<0.05) and MLECs by 33±4.5% (p<0.05). Furthermore, addition of the AM antagonist, AMA (1 uM), reduced the basal growth of HPSCs by 21±5.3% (p<0.05), HUVECs by 21±0.4% (p<0.05) and MLECs by 25±5.1% (p<0.05) further supporting an autocrine role of AM on these cells. To further examine the effects of AM and we conducted *in vitro* angiogenesis assays. AM stimulated polygon formation in HUVECs (Fig. 1F) and in MLECs (Fig. 1G) at 6 hours compared to control cultures, and AMA blocked the AM mediated effects of polygon formation.

### The effects of AM on HPSC, HUVEC and MLEC cells are mediated via the ADMR receptor

**Figure 2:**
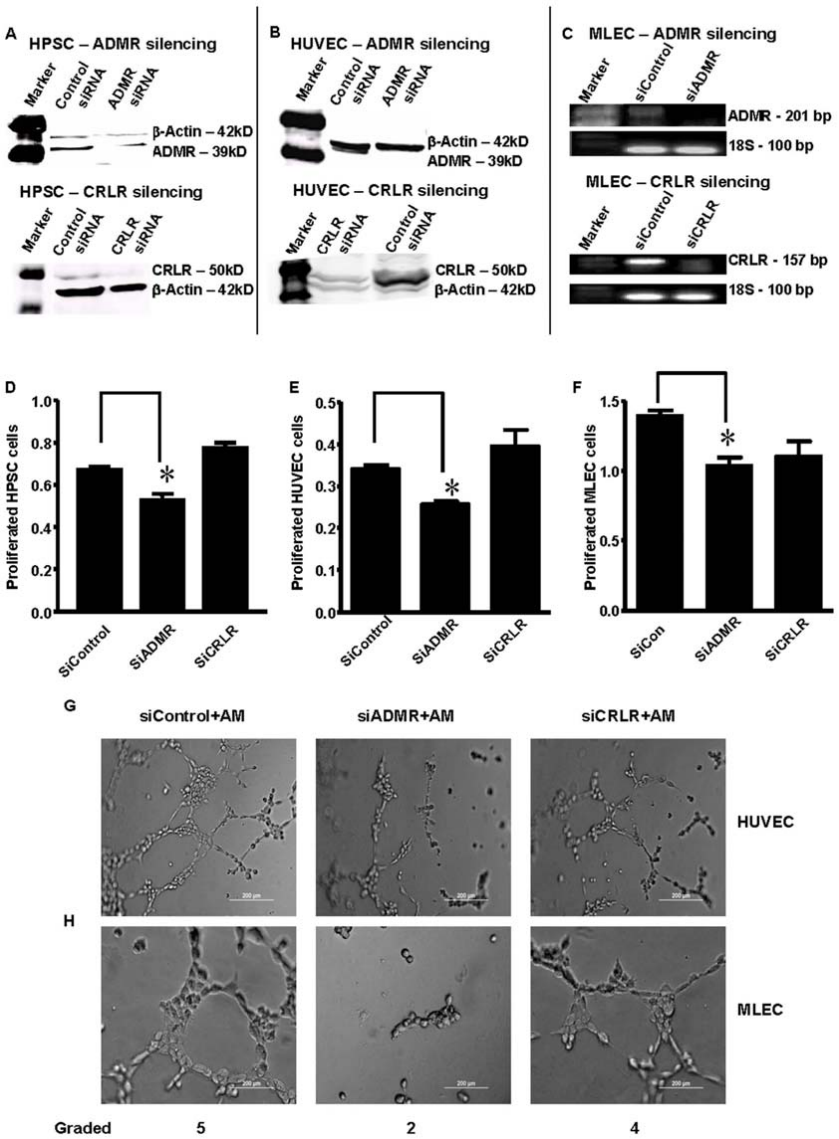
Autocrine effects of AM on HPSC and endothelial cells are mediated via ADMR but not CRLR. (A) HPSC and (B) HUVEC cells were transiently transfected with human siRNAs and (C) MLEC cells were transfected with mouse siRNAs (siControl, siADMR, or siCRLR). After 72 hours cells were harvested and protein or RNA was extracted. Western blotting using human antibodies shows the extent of silencing of human ADMR and CRLR in HPSC and HUVEC cells, and RT-PCR using mouse primers shows the effects of mouse siADMR and siCRLR on MLECs. β-Actin served as a loading control for western blotting and 18S primers served as a loading control for RT-PCR. Full length gels are presented in Figure S1. The effects on proliferation of siRNA mediated silencing of receptors are shown on (D) HPSC, (E) HUVEC, and (F) MLEC cells. Cells were transfected with their respective human or mouse siRNAs (siControl, siADMR, or siCRLR) for 24 hours then proliferation was estimated by MTS assay after an additional 48 hours for HPSC and HUVEC cells and 96 hours for MLECs. Silencing of ADMR but not CRLR caused a significant reduction of the proliferation of these cells. Data shown are means +/− SEM (*p<0.05). *In vitro* angiogenesis assay with (G) HUVEC and (H) MLEC cells. Cells were plated on matrix gel 24 hours after transfection with their respective human or mouse siRNAs (siControl, siADMR, or siCRLR). Cells were then treated with AM (200 nM) for 6 hours and examined microscopically. The extent of tube formation was evaluated as per the manufacturer's instruction. Representative micrographs are shown. ADMR silencing completely blocked tube formation, while CRLR silencing only partially reduced tube formation.

There are two potential receptors that respond to AM, ADMR and CRLR. We previously reported that human pancreatic cancer cells express exclusively ADMR while HPSC and HUVEC cells expressed both AM receptors [3]. In the current study, we observed that MLECs also express both receptors. To determine the relative importance of these receptors we silenced them independently on these three cell lines using siRNA techniques. Transfection with siRNA significantly reduced the levels of either ADMR or CRLR on HPSC (Fig. 2A), HUVEC (Fig. 2B) and MLECs (Fig. 2C) as compared to control siRNAs. HPSC, HUVEC and MLECs silenced with siRNA for either ADMR or CRLR were examined in growth assays where cell numbers were estimated using the MTS method. Silencing of ADMR, but not CRLR, significantly reduced the growth of all of these cell types. Silencing of ADMR reduced the growth of HPSC by 21±3.4% (p<0.05) (Fig. 2D), HUVECs by 24±1.8% (p<0.05) (Fig. 2E) and MLECs by 26±4.3% (p<0.05) (Fig. 2F). Silencing of ADMR on HUVECs (Fig. 2G) and MLECs (Fig. 2H) completely abolished AM mediated tube formation, while silencing of CRLR partially reduced tube formation. These data collectively suggest that the autocrine effects of AM on HPSC, HUVEC and MLEC cells are mediated primarily via the receptor ADMR although CRLR may also have some effects.

### Silencing of ADMR on pancreatic cancer cells reduced tumor growth and metastasis *in vivo*

**Figure 3:**
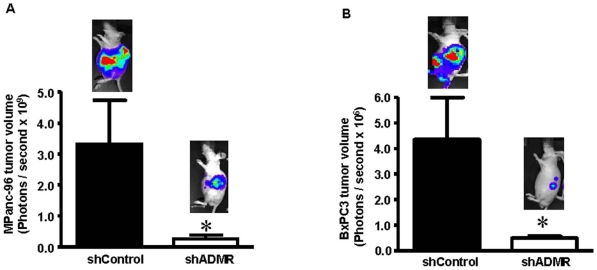
Effect of ADMR on tumor growth *in vivo*. Orthotopic tumors were developed with MPanc96 (A) or BxPC3 (B) cells stably silenced with shADMR and expressing the luciferase gene. After 4 weeks, the tumor volume was estimated by bioluminescence imaging and showed a significant reduction between ADMR and shControl vector bearing tumors from both cell types. Data shown are means +/− SEM for 10 animals per group. (*p<0.05) Bioluminescence images of representative mice are also provided.

To examine the effects of ADMR silencing on cancer cells, we observed the growth of orthotopic tumors formed from two different pancreatic cancer cell lines transfected with either shADMR or shControl. Tumor volumes were measured after 4 weeks by bioluminescence imaging. The tumor volume was reduced by 92±0.5% in ADMR silenced MPanc96 tumors when compared to control vector bearing tumors (p<0.05) (Fig. 3A). Likewise, BxPC3 tumor volume was significantly reduced by 83±0.6% after ADMR silencing compared to control tumors (p<0.05) (Fig. 3B). In both cases, there was also a reduction in lung and liver metastasis and a reduction in peritoneal dissemination. However, because ADMR silencing reduced the tumor volume; any reduction in metastasis may have been due to the general reduction in tumor volume.

### Silencing of ADMR on pancreatic cancer cells reduced metastasis *in vivo*

**Figure 4:**
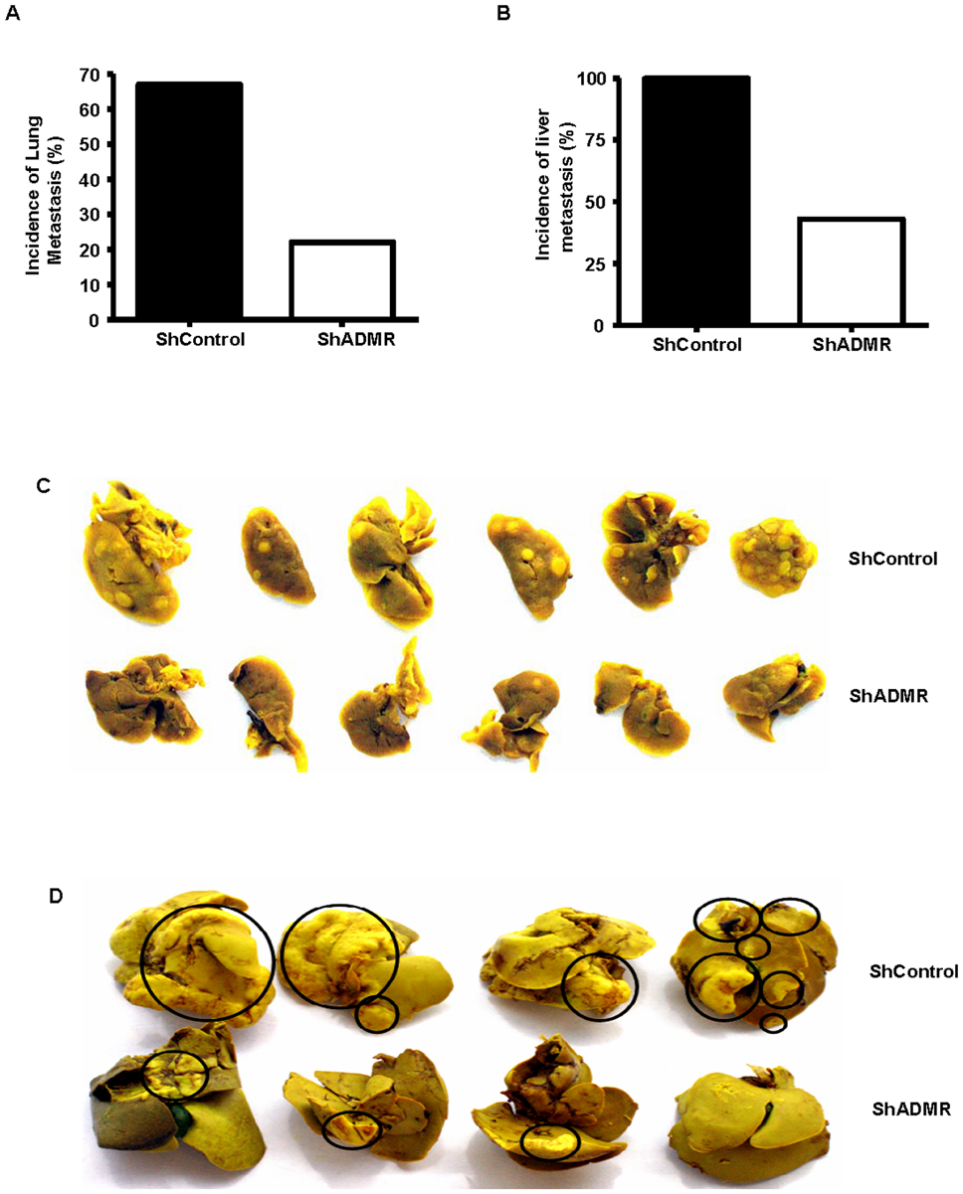
Effects of ADMR on metastasis. MPanc96 cells bearing luciferase gene stably transfected with shControl or shADMR cells were injected into the tail vein to measure lung metastasis and into the spleen to measure liver metastasis. Animals bearing ADMR silenced cells showed reduction in the incidence of lung (A) and liver metastases (B) as measured by bioluminescence imaging. After 6 weeks mice were sacrificed and lung and liver were also excised and examined grossly and histologically. Stable silencing of ADMR reduced the number of metastatic foci on lung and liver when compared to shControl. Representative pictures show the reduction in number of metastatic foci in lungs (C) and livers (D).

To directly evaluate the role of ADMR in pancreatic cancer lung and liver metastasis, we conducted separate studies in *nude* mice by tail vein and splenic injection, respectively. ADMR silencing reduced the incidence of lung metastasis (ShControl - 67% Vs ShADMR – 22%) as measured by bioluminescence imaging (Fig. 4A). Furthermore, the presence of metastasis detectable using bioluminescence imaging was postponed from the 2^nd^ week in control animals to 5^th^ week in shADMR bearing cells. Excised lungs after fixation in Bouin's solution showed reduction in the number of lung metastatic foci as shown in the representative image (Fig. 4C). The effects of ADMR silencing on liver metastasis were similar to those on lung metastasis. ADMR silencing reduced the incidence of liver metastasis (ShControl - 100% Vs ShADMR – 43%) (Fig. 4B). The presence of detectable metastases was postponed from the 2^nd^ week until the 5^th^ week. Reduction in the number of liver metastatic foci is shown as a representative image (Fig. 4D). These data indicate that AM can act as a metastasis inducer of pancreatic cancer cells and these effects of AM on cancer cells are mediated via the receptor ADMR.

### I*n vivo* targeting of ADMR using systemic delivery of siRNAs is highly effective at reducing tumor growth

**Figure 5:**
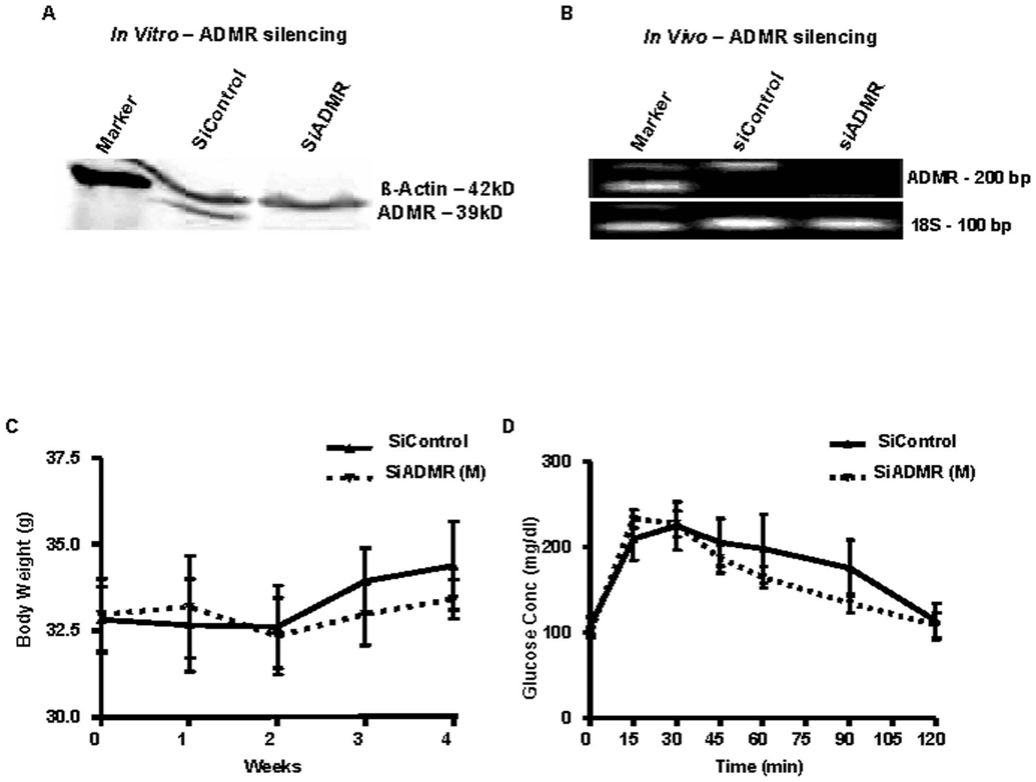
Effect of ADMR silencing in tumor microenvironment cells *in vivo*. (A) MPanc96 cells were transfected with siControl or siADMR and after 72 hours western blotting was conducted. SiADMR significantly reduced expression of ADMR on MPanc96 cells. The same blot was probed for β-Actin, which served as loading control. (B) Athymic nude mice were treated with DOPC nanoliposome coupled siControl or siADMR (mouse) (10 ug per animal i.p. twice a week for four weeks), then sacrificed and the pancreas was analyzed ADMR by RT-PCR using the mouse ADMR primers. ADMR was almost completely silenced by siADMR treatments. 18S served as loading control. Full length gels are presented in Figure S2. (C) Body weight of mice treated with DOPC nanoliposome coupled siControl or siADMR did not show any difference after a period of four weeks. (D) Glucose tolerance test (GTT) was performed on these mice by injecting 1.5 mg glucose/g body weight i.p. Systemic silencing of ADMR did not affect glucose levels when compared to DOPC nanoliposome coupled siControls.

Our data support a role for ADMR in the effects of AM on pancreatic cancer cells. However, AM has physiologic functions such that inhibition of one of its receptors might cause harmful effects on overall health. To analyze the effects of targeting these receptors systemically *in vivo*, we silenced ADMR on both human cancer cells and on mouse tissue using *in vivo* delivery of siRNA in DOPC nanoliposomes. We first examined the efficiency of using targeted siRNAs to reduce ADMR levels in human pancreatic cancer cells by western blot analysis of ADMR levels in MPanc96 cells. Transient transfection of siADMR completely silenced the ADMR expression in MPanc96 cells (Fig. 5A). Silencing of ADMR did not influence the cellular levels of AM as measured by ELISA (data not shown). Therefore, this siRNA was highly effective at silencing ADMR expression in human cells. However, these siRNAs had no effect on ADMR expression in NIH3T3 mouse cells (data not shown).

To silence ADMR in the mouse, we developed DOPC nanoliposome coupled siControl and siADMR (mouse specific). We examined the effectiveness and the potential toxicity of delivering these siRNA (10 ug per animal i.p. twice a week for four weeks). We observed that delivery of DOPC nanoliposome coupled mouse siADMR for a period of four weeks led to a complete silencing of ADMR in the pancreas as analyzed by RT-PCR (Fig. 5B). In contrast, there was no decrease in ADMR expression in animals treated with DOPC nanoliposome coupled siControl. Importantly, silencing of ADMR in the mouse by i.p. delivery of DOPC nanoliposomes did not have any noticeable effect on the body weight (Fig. 5C), water intake or physical activity of the mice. Since AM is said to have inhibitory effects on insulin secretion [6], blood glucose levels were measured to see if any changes occurs on systemic silencing of ADMR in mouse. GTT showed no changes on DOPC nanoliposome coupled mouse siADMR injected animals when compared to DOPC nanoliposome coupled siControl animals (Fig. 5D). These observations suggest that silencing of ADMR, at least for four weeks, was not toxic or overtly deleterious.

**Figure 6:**
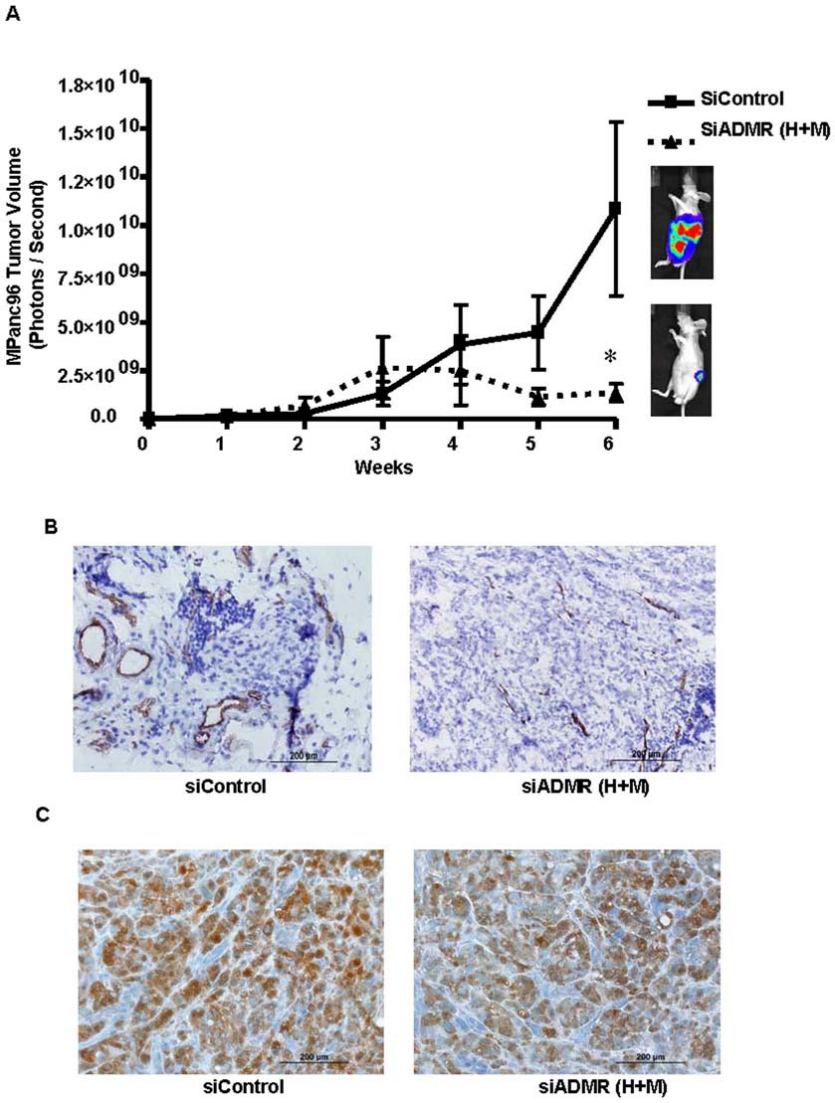
Effects of systemic delivery of DOPC nanoliposome coupled human and mouse siADMR *in vivo*. (A) *In vivo* targeting of ADMR using DOPC nanoliposome coupled human and mouse siADMR in combination. SiRNAs were delivered (each 10 ug per animal i.p. twice a week) to *athymic nude* mice bearing human pancreatic cancer cells (MPanc96 with luciferase gene) and monitored for a period of six weeks. These treatments caused a significant reduction in tumor volume as measured by bioluminescence imaging. Representative pictures of mice show the bioluminescence images of treated and untreated pancreatic cancer. (B) Tissues from orthotopic tumors developed with MPanc96 cells and treated with siControl or siADMR (human and mouse in combination) were processed immunohistochemically for CD31 staining. SiControl treated cells showed open blood vessels, while siADMR treated cells showed constricted or collapsed blood vessels. Shown are representative micrographs. (C) Immunohistochemical staining of VEGF showed no differences between siControl or siADMR treated tumors.

Next we examined the effectiveness of delivering a combination of DOPC nanoliposome coupled siRNAs against human and mouse ADMR to simulate systemic therapies that might be utilized in humans. The DOPC nanoliposome coupled siRNAs were delivered to animals with orthotopic tumors formed from human MPanc96 cells and tumor growth was monitored by bioluminescence imaging for a period of six weeks (Fig. 6A). Silencing of ADMR in both cancer cells and the tumor microenvironment in combination resulted in a significant reduction in tumor volume by 88±0.4% (p<0.05) at the end of six weeks when compared to its control. Analysis of the effects of ADMR silencing on endothelial cells *in vivo* was done using CD31 staining on tissue sections from mice bearing DOPC nanoliposome coupled siControl or siADMR. Silencing of ADMR resulted in significant reduction in open blood vessels when compared to their control tissues (Fig. 6B). We also analyzed the effects of silencing ADMR on VEGF production on these sections. Silencing of ADMR did not alter VEGF production (Fig. 6C).

## Discussion

In the current study, we evaluated the role of AM receptors in tumor cells and cells in the tumor microenvironment and examined the effectiveness of inhibiting ADMR as a potential therapeutic target. We previously reported that AM is over-expressed in pancreatic cancer and stimulates cancer cells via an autocrine loop resulting in increased tumor growth [3]. Other studies have indicated that AM also promotes tumor growth by stimulation of angiogenesis through effects on endothelial cells within the tumor microenvironment [10]. Taken together, these observations suggested that AM might be a useful target for cancer therapy. However, AM has complex physiological roles mediated by its actions on at least two types of G-protein-coupled receptors, CRLR [26] and ADMR [19]. For this reason, there is a concern that systemic inhibition of AM may have significant and possibly harmful effects on normal physiology. Therefore, rather than targeting AM itself, we were interested in the possibility of targeting one of its receptors. Currently little is known about the relative contributions of ADMR or CRLR to the physiological or pathophysiological actions of AM. In the current study, we found that ADMR is the primary receptor responsible for the growth stimulatory effects of AM on pancreatic cancer, HPSCs and endothelial cells and that ADMR silencing by systemic delivery of siRNA was feasible and effective against pancreatic cancer without obvious detrimental effects to overall health.

In this study, we showed for the first time that both HPSC and endothelial cells secreted AM, similar to what we previously observed for cancer cells [3]. Also in this study, treatment with a short peptide of AM, which acts as an antagonist, reduced basal proliferation of each of these cell types. These data suggest the presence of an autocrine loop. We also observed that exogenous addition of AM stimulated the growth of each of these cell types and stimulated *in vitro* angiogenesis in cultures of endothelial cells. It has previously been reported that AM treatment stimulates endothelial cell proliferation and *in vitro* angiogenesis [12], [27]. It has also previously been observed that AM is secreted by human hepatic stellate cells, which are similar to pancreatic stellate cells, and acts in an autocrine manner to regulate contractility of these cells [28]. Therefore, AM is present and likely secreted by several cell types that constitute the tumor microenvironment.

AM has two potential receptors, ADMR and CRLR. We were interested to determine whether both receptors were important for the ability of AM to stimulate pancreatic tumor growth or whether one receptor might be more important. We previously observed that pancreatic cancer cells express exclusively ADMR [3]. However, in that study we did not determine whether this receptor was required for the effects of AM on the cancer cells. Furthermore, currently the receptor involved in the effects of AM on endothelial cells or stellate cells is unknown. It has been reported that endothelial cells express CRLR [27] and it has been suggested that CRLR is the main mediator of the effects of AM on the vasculature [29]. Therefore, we examined the importance of the different receptors by silencing them individually on each of the cell types. We found that silencing ADMR reduced the proliferation of HPSC, HUVEC and MLEC cells *in vitro* while silencing of CRLR had no effect. ADMR was also primarily responsible for the angiogenic effects of AM on HUVECS and MLECs, while CRLR had lesser effects. Previously it was reported that an anti-CRLR antibody reduced the angiogenic functions of AM on endothelial cells [27], [30]. However, in that study they did not evaluate the effects of ADMR. Our data clearly indicate the primary importance of ADMR on pancreatic cancer cells and cells resembling the stellate and endothelial cells found in the pancreatic tumor microenvironment.

Having identified ADMR as a critical AM receptor for pancreatic tumors, we tested the effect of silencing ADMR *in vivo* as a potential approach to pancreatic cancer therapy. We first examined the effect of silencing ADMR specifically in cancer cells by stably silencing ADMR in human pancreatic cancer cells in vitro and then using them to develop orthotopic tumors. We observed a dramatic reduction on tumor volume after silencing ADMR in two different pancreatic cancer cell lines. These dramatic effects emphasized the importance of the ADMR receptor and the AM autocrine loop in pancreatic cancer cells.

We also observed a reduction in metastasis after silencing ADMR. However, because of the large effect of ADMR silencing on primary tumor growth, it was difficult to determine if this involved more than simply reduced cancer cell growth. To determine whether the reduction in metastasis was independent of the reduced tumor growth, we examined the behaviour of the ADMR silenced cells in lung and liver metastasis studies. By injection of cancer cells into the tail vein, the cells are carried to various organs but tend to form colonies in the lung. Similarly, injection of cancer cells into the spleen leads to the development of metastases in the liver. The number of colonies formed in these assays does not depend on the growth rate of the cells. Using sensitive bioluminescence imaging we observed a highly significant reduction in the incidence of metastases as well postponement on the presence of detectable metastases in both lung and liver using ADMR silenced cells. Reduction in number of lung and liver metastatic foci were also seen on silencing ADMR *in vivo*. These studies supported AM as a metastatic factor apart from also being a growth inducer and potent angiogenic factor.

To develop the idea of targeting ADMR therapeutically, we investigated the effects of systemic delivery of siRNA using liposomes. Initially we were concerned that the silencing of this receptor might prove deleterious. Previously it was reported that CRLR knock-out mice were embryonically lethal [31]. However, when we silenced ADMR by systemic delivery of DOPC nanoliposome coupled siRNA to mouse ADMR we did not observe any effects on body weight, feeding behavior, activity or blood glucose levels of the animals, despite highly effective silencing of ADMR in the pancreas. Thus, tentatively it appears that systemic ADMR silencing is not overtly harmful. Clearly, full toxicology studies will be required before moving this treatment forward to human trials. Notably, unlike typical siRNA studies, in the current study ADMR was silenced on both the cancer cells and systemically by using siRNAs of both human and mouse ADMR. Silencing of only the human form does not test the systemic effects of siRNA treatment as would occur if this treatment was translated to the clinic. We observed that treatment of the mice with the combination of both human and mouse siRNA to ADMR greatly inhibited tumor growth and angiogenesis *in vivo* without obvious harmful effects.

The reduction of angiogenesis associated with ADMR silencing was not associated with a reduction in VEGF production. This is in keeping with previous observations suggesting that AM does not act through VEGF stimulation in the process of angiogenesis. It was previously shown that down regulation of VEGF or its receptor, VEGFR-2, could not suppress AM induced angiogenesis [30]. Interestingly, an AM antagonist was able to inhibit VEGF induced blood flow [32]. Our data suggests that AM acts as an VEGF-independent angiogenic factor and a reduction in AM's function in cancer cells and the adjacent tumor microenvironment cells results in reduced neoangiogenesis.

Thus, this study collectively delineated the autocrine effects of AM in pancreatic cancer and identified ADMR as the critical receptor through which these effects are mediated on both tumor cells and cells within the tumor microenvironment. Because the effects of ADMR silencing on pancreatic cancer cells were so dramatic, we expect that this is the major site of action of the *in vivo* silencing experiments. Importantly, we showed that silencing ADMR *in vivo* greatly reduced tumor growth without overt deleterious effects. These data generally support the suggestion that ADMR may be a potent therapeutic target in pancreatic cancer.

## Supporting Information

Figure S1 Silencing of ADMR or CRLR on HPSCs, HUVECs, or MLECs. (A) HPSC and (B) HUVEC cells were silenced with respective human siRNAs (5nM) for 72 hours. Western blotting was conducted for either ADMR or CRLR using human antibodies and the same blots were probed for β-Actin, which served as loading control. (C) MLEC cells were transfected with mouse siRNAs against ADMR and CRLR and after 72 hours total RNA was isolated and RT-PCR was conducted with respective mouse primers and with 18S, which served as loading control.
(0.73 MB TIF)

Figure S2 Western blot of MPanc96 cells showing the silencing effect of siADMR. MPanc96 cells transfected with siRNAs (5nM) (siControl, siADMR or siCRLR) showed a significant silencing of ADMR after 72 hours. The same blot was probed for β-Actin which served as loading control (In vitro silencing). Athymic nude were treated with DOPC nanoliposomes coupled with siControl or siADMR (mouse) (10 ug per animal i.p. twice a week for four weeks) and were sacrificed and the level of endogenous ADMR was evaluated. RT-PCR using mouse ADMR primers showed the complete silencing of ADMR after delivery of DOPC nanoliposome coupled mouse siADMR as compared to siControl delivered animals. 18S served as loading control (In vivo silencing).
(0.24 MB TIF)
