# Supplementary figures and images for: The ADMR Receptor Mediates the Effects of Adrenomedullin on Pancreatic Cancer Cells and on Cells of the Tumor Microenvironment
Source: PLoS One. 2009 Oct 22;4(10):e7502. doi: 10.1371/journal.pone.0007502 (PMC2760778; doi:10.1371/journal.pone.0007502)

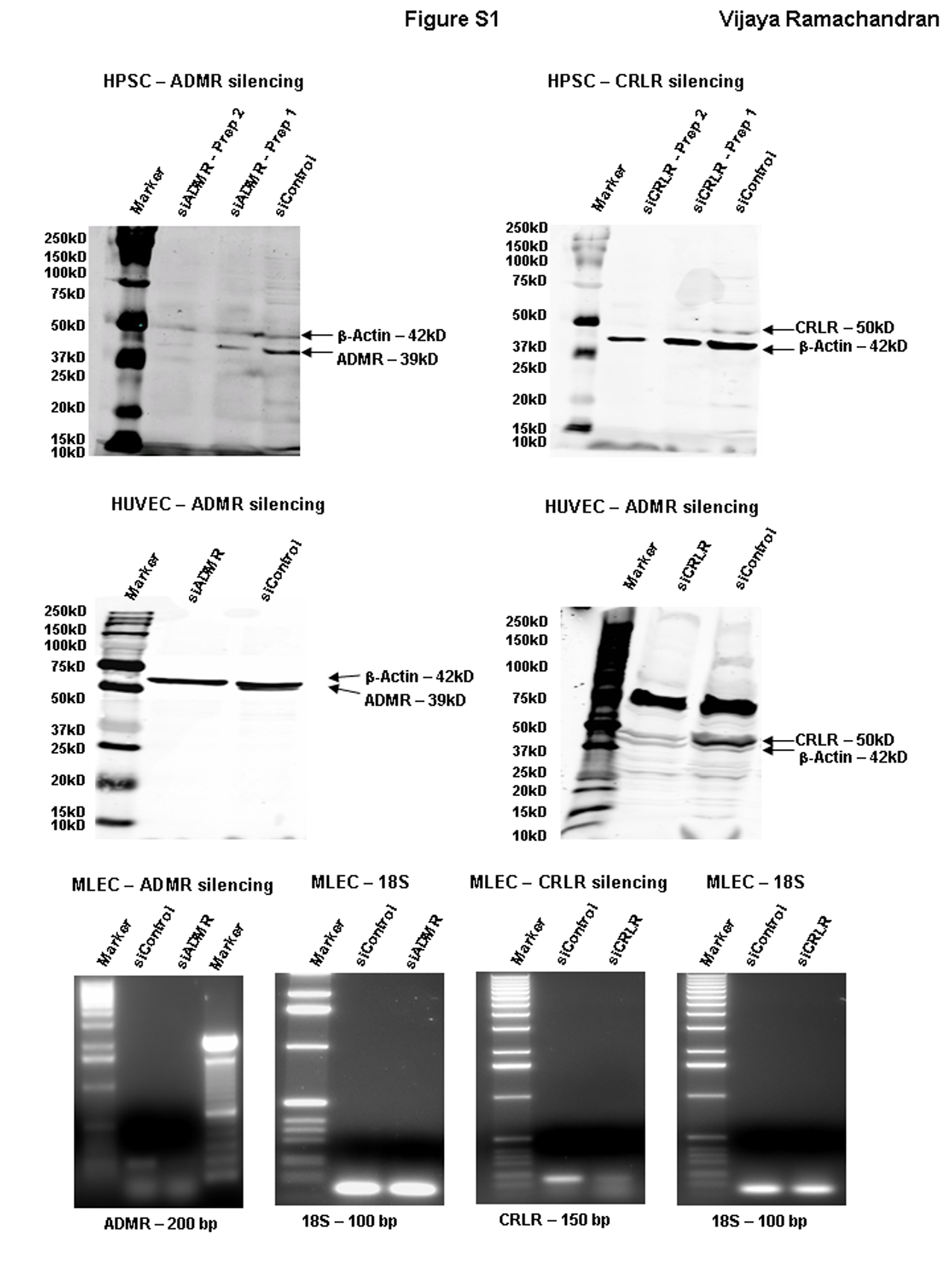

Supplement: Figure S1 — Silencing of ADMR or CRLR on HPSCs, HUVECs, or MLECs. (A) HPSC and (B) HUVEC cells were silenced with respective human siRNAs (5nM) for 72 hours. Western blotting was conducted for either ADMR or CRLR using human antibodies and the same blots were probed for β-Actin, which served as loading control. (C) MLEC cells were transfected with mouse siRNAs against ADMR and CRLR and after 72 hours total RNA was isolated and RT-PCR was conducted with respective mouse primers and with 18S, which served as loading control. (0.73 MB TIF) [file pone.0007502.s001.tif]

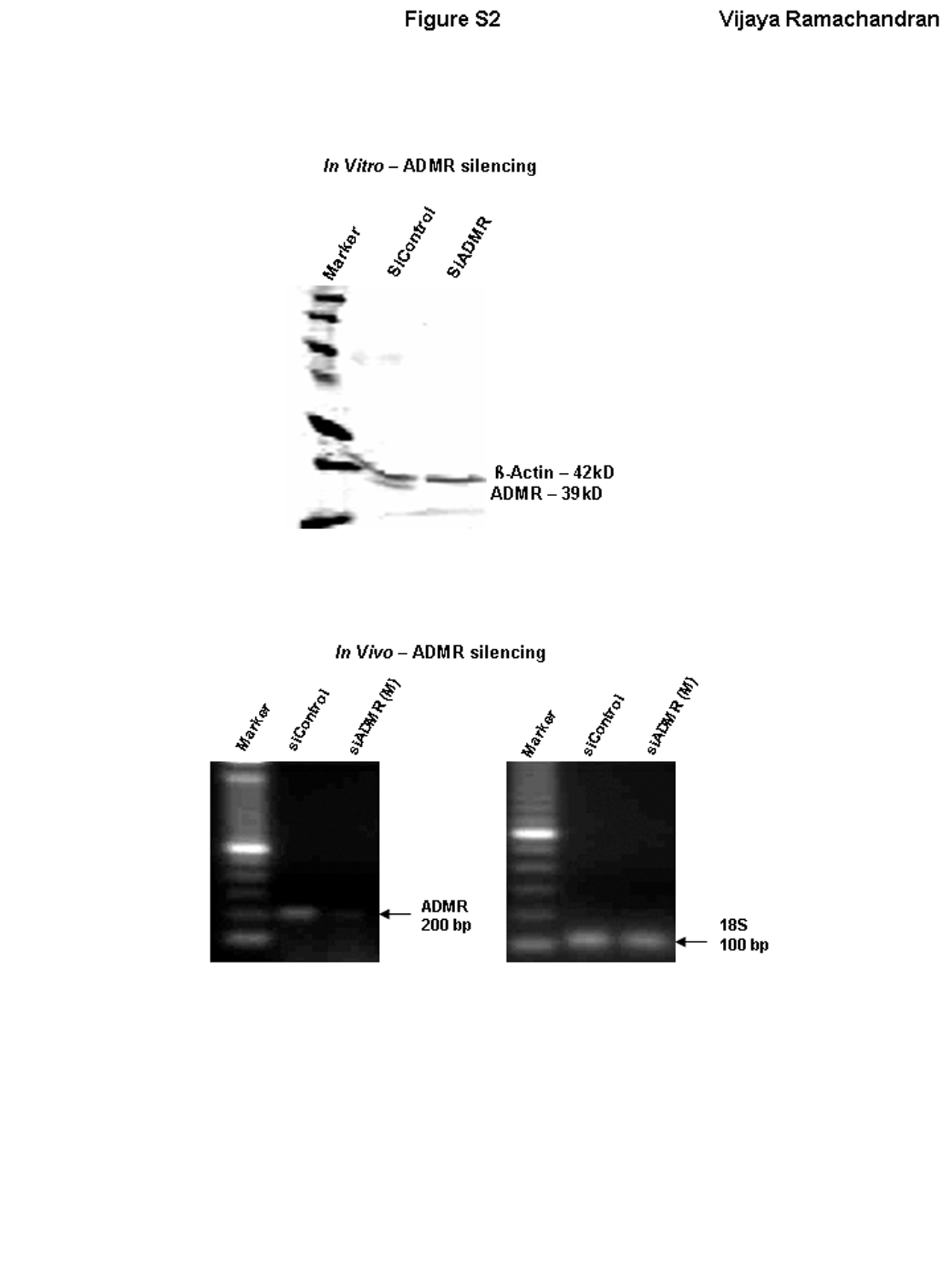

Supplement: Figure S2 — Western blot of MPanc96 cells showing the silencing effect of siADMR. MPanc96 cells transfected with siRNAs (5nM) (siControl, siADMR or siCRLR) showed a significant silencing of ADMR after 72 hours. The same blot was probed for β-Actin which served as loading control (In vitro silencing). Athymic nude were treated with DOPC nanoliposomes coupled with siControl or siADMR (mouse) (10 ug per animal i.p. twice a week for four weeks) and were sacrificed and the level of endogenous ADMR was evaluated. RT-PCR using mouse ADMR primers showed the complete silencing of ADMR after delivery of DOPC nanoliposome coupled mouse siADMR as compared to siControl delivered animals. 18S served as loading control (In vivo silencing). (0.24 MB TIF) [file pone.0007502.s002.tif]
